# Sinapic acid attenuates cyclophosphamide-induced liver toxicity in mice by modulating oxidative stress, NF-κB, and caspase-3

**DOI:** 10.22038/IJBMS.2023.68579.14960

**Published:** 2023

**Authors:** Shiva Rezaei, Seyed Jalal Hosseinimehr, Mehryar Zargari, Abbasali Karimpour Malekshah, Mansooreh Mirzaei, Fereshteh Talebpour Amiri

**Affiliations:** 1 Department of Anatomy, Faculty of Medicine, Molecular and Cell Biology Research Center, Mazandaran University of Medical Sciences, Sari, Iran; 2 Student Research Committee, Faculty of Medicine, Mazandaran University of Medical Sciences, Sari, Iran; 3 Department of Radiopharmacy, Faculty of Pharmacy, Mazandaran University of Medical Sciences, Sari, Iran; 4 Department of Biochemistry, Faculty of Medicine, Mazandaran University of Medical Sciences, Sari, Iran

**Keywords:** Apoptosis, Cyclophosphamide, Liver injury, Inflammation, Oxidative stress, Sinapic acid

## Abstract

**Objective(s):**

Cyclophosphamide (CP) as an antineoplastic drug is widely used in cancer patients, and liver toxicity is one of its complications. Sinapic acid (SA) as a natural phenylpropanoid has anti-oxidant, anti-inflammatory, and anti-cancer properties.

**Materials and Methods:**

The purpose of the current study was to determine the protective effect of SA versus CP-induced liver toxicity. In this research, BALB/c mice were treated with SA (5 and 10 mg/kg) orally for one week, and CP (200 mg/kg) was injected on day 3 of the study. Oxidative stress markers, serum liver-specific enzymes, histopathological features, caspase-3, and nuclear factor kappa-B cells were then checked.

**Results:**

CP induced hepatotoxicity in mice and showed structural changes in liver tissue. CP significantly increased liver enzymes and lipid peroxidation, and decreased glutathione. The immunoreactivity of caspase-3 and nuclear factor kappa-B cells was significantly increased. Administration of SA significantly maintained histochemical parameters and liver function enzymes in mice treated with CP. Immunohistochemical examination showed SA reduced apoptosis and inflammation.

**Conclusion:**

The data confirmed that SA with anti-apoptotic, anti-oxidative, and anti-inflammatory activities was able to preserve CP-induced liver injury in mice.

## Introduction

Cyclophosphamide (CP) is an alkylating factor mainly used in chemotherapy of patients with cancer, autoimmune diseases, and organ transplants (1). However, despite the wide application of CP in the clinic, the adverse effects of this drug on viscera, especially liver tissue, are high (2). The main mechanisms of action of CP in the induction of hepatotoxicity are oxidative stress and activation of inflammatory cascade reaction (3). Phosphoramide mustard and acrolein originate from the metabolization of CP in the liver by microsomal cytochrome P450 (4), which increases reactive oxygen species (ROS), weakens the liver’s anti-oxidant defense system (5), stimulates lipid peroxidation (6), and activates nuclear factor-kappa B (NF-κB) (7). Also, oxidative stress and inflammation can lead to hepatocyte apoptosis. Therefore, the use of anti-oxidants and anti-inflammatory agents may play a role in maintaining or ameliorating liver damage (8).

Sinapic acid (SA) is one of the most common hydroxycinnamic acids that occur widely in the plant kingdom and has been identified in various fruits, vegetables, cereal seeds, oilseed products, several spices, and pharmaceutical plants (9). It has been reported as a powerful anti-oxidant in many studies (10). Animal studies have shown the therapeutic activity of SA such as anti-oxidant, anti-inflammatory, anti-cancer, and anti-anxiety activity (9). SA suppressed oxidative stress and proinflammatory cytokine expression through NF-κB inactivation (11). It protected the heart against isoproterenol (12), the kidney against gentamicin, and the liver against arsenic (13) and carbon tetrachloride (14). SA has a cytotoxic effect on a human laryngeal carcinoma cell line (15). Although several biological and pharmacological activities of SA have been investigated, there is no study on the effect of SA against liver damage caused by CP.

Considering the above properties of SA, this research was designed to investigate the hepatoprotective effect of SA on CP-induced liver injury in mice, with an emphasis on anti-oxidant, anti-inflammatory, and anti-apoptotic assays.

## Materials and Methods

***Materials***

SA was procured from Sigma-Aldrich (St. Louis, MO, USA) and cyclophosphamide from Baxter Company (Germany). It was suspended in distilled water containing 1% carboxymethyl cellulose (CMC).

***Experimental design***

Adult male BALB/c mice (25–30 g) were procured from the Animal Research Center of Mazandaran University of Medical Sciences, Sari, Iran. The present study was approved by the Institutional Animal Ethics Committee of Mazandaran University Medical Sciences (ID: IR.MAZUMS.REC.1399.6883). The animals were adapted to the laboratory environment for a week before the beginning of the studies and were kept in standard temperature conditions with 23 ± 2 degrees Celsius, 55 ± 5% humidity, 12 hr of light and darkness, and free access to water and food. The mice were then divided into four groups:

**Control group**: animals received CMC orally daily for one week.

**SA groups**: animals were treated with SA at two doses of 5 and 10 mg/kg/day orally for a week. 

**CP group**: hepatotoxicity developed by CP (200 mg/kg/day) by intraperitoneal injection of a single dose on day 3 of the study.

**Figure 1:**
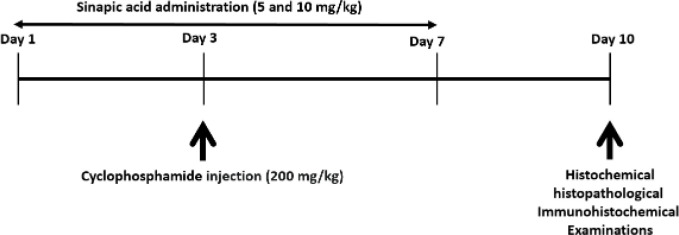
Diagram of the study design of the effect of cyclophosphamide and sinapic acid on liver tissue: Time of drug administration and time of tests

**SA+CP groups**: mice were treated with CP (200 mg/kg/day) and SA at two doses of 5 and 10 mg/kg/day; respectively. CP dose was selected according to our previous study (16) and SA according to other studies(13). An outline of the design of this research was drawn in Figure 1.

***Samples collection***

Three days after taking the last medicine, the mice were anesthetized with ketamine (50 mg/kg) and xylazine (5 mg/kg), then blood was collected and centrifuged, and serum was separated and stored for biochemical analysis. After that, the liver was immediately dissected and washed in phosphate buffer saline (PBS), weighed, and then part of it freshly frozen and stored at −80 °C for analysis of liver enzymes. And another part of it was fixed in 10% buffer formalin solution to evaluate the histopathological and immunohistochemical test. 

***Serum biochemical analysis of liver enzymes***

Serum alanine transaminase (ALT), aspartate transaminase (AST), and alkaline Phosphatase (ALP) activities were estimated in accordance with the manufacturer’s instructions (1 400 018; AST, 1 400 019; ALT and 12,201; ALP). The liver enzyme level was recorded as U/l. 

***Tissue biochemical analysis***

Malondialdehyde (MDA) content was determined by measuring thiobarbituric acid (TBA) in the liver tissues. First, it was homogenized, then samples were mixed with 1 ml of 0.6 % 2-thiobarbituric acid, 3 ml of 1% phosphoric acid, and 0.1 ml of distilled water. The mixture was boiled (45 min) in a water bath, then the mixture was cooled, later 4 ml of n-butanol was added to extract the cold thiobarbituric acid reactants. Then, samples were centrifuged at 3500 rpm for 10 min to separate the butanol layer. The n-butanol layer optical density was determined by spectrophotometry (UV-1601 PC, Shimadzu, Japan). A standard curve of MDA was created. MDA concentration was expressed as nmol/g tissue.

The content of the glutathione (GSH) in the liver tissue was calculated by a spectrophotometer with 5,5’-dithiobis-2-nitrobenzoic acid (DTNB) as an indicator at 412 nm and expressed as μmol/g tissue.

***Histopathological assay***

The liver specimens were fixed in 10% neutral-buffered formalin solution for 24 hr, dehydrated in the graded series of ethanol solutions, cleared in xylene, embedded in paraffin, and then sections with 5 mm thickness were prepared by using a rotary microtone and stained with H&E (hematoxylin and eosin). The liver slides were evaluated blindly by a histologist using light microscopy at 400X magnification (Olympus light microscope, Japan). For the semi-quantitative evaluation of samples, histological photomicrographs were investigated by the liver injury scoring system. In the present study, the amount of degeneration, sinusoidal dilatation, inflammatory cell infiltration, congestion, proliferation of Kupffer cells, and cytoplasmic vacuolization were calculated. For each mouse, five sections, and in each section, 10 fields were randomly estimated. Data were scored as 0 (normal), 1 (mild), 2 (moderate), or 3 (severe) (17).

***Immunohistochemical assay***

Immunohistochemical examination for caspase-3 (as apoptotic marker) and NF-κB was performed according to the guidance kit company (Abcam, USA). Sections 5 µm thick were deparaffinized with xylene and rehydrated in alcohol series, endogenous peroxidase activities were blocked by 0.3% H_2_O_2_ in methanol (30 min), and proteins were blocked with protein blocker (10 min). Then, sections were incubated with primary antibodies (anti-rabbit polyclonal antibody, 1:75, v/v, Abcam, lat: GR224831-2 and 831054 ab7970-1) overnight. After incubation with horseradish peroxidase-conjugated secondary antibody (Mouse and Rabbit Specific HRP/DAB, Abcam, Lat: GR2623314-4) for 2 hr, sections were incubated with diaminobenzidine tetrahydrochloride for 5 min (6). The slides were then dehydrated and mounted. Primary antibody was omitted for negative controls. For the quantitative analysis, immunohistochemical micrographs were evaluated by densitometry using Mac Biophotonics ImageJ 1.41a software. The intensity of positive staining was determined as the ratio of the stained area to the entire field evaluation. 

***Statistical analysis***

Results were expressed as mean ± SD. All statistical comparisons were performed by one-way analysis of variance (ANOVA) followed by Tukey’s post-test. *P*-value less than 0.05 (*P*<0.05) was considered statistically significant (Prism software, USA).

## Results

***Serum parameters of liver function***

Table 1 shows the analysis of the activity of liver enzymes such as ALT, AST, and ALP to determine the preventive effect of SA on liver tissue damage caused by CP. ALT and AST levels were significantly increased in CP-treated mice compared with the control group (*P*<0.001). Oral administration of SA at both 5 and 10 mg/kg doses significantly ameliorated liver enzymes in CP-treated mice. SA at the 10 mg/ kg dose did not show any change in AST and ALP levels. SA at either 5 or 10 mg/kg dose did not modify liver functional markers in mice.

***Liver oxidative stress markers***

**Figure 2:**
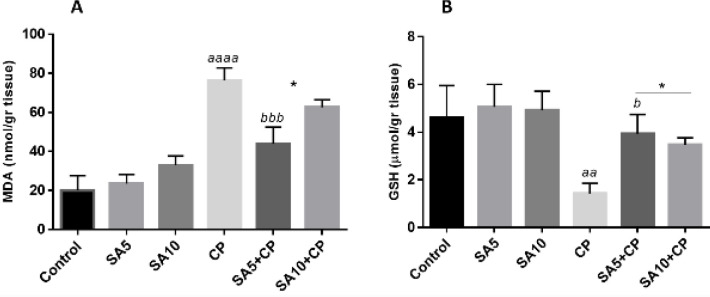
Oxidative stress biomarkers. All charts are presented as mean ± SD. a, significant vs control; b, significant versus CP. cyclophosphamide (CP); Sinapic acid (SA)

Figure 2 illustrates the hepatic lipid peroxidation and glutathione contents in all groups. The level of MDA was increased in CP-treated animals compared with the control group (*P*<0.0001). Oral administration of SA (5 mg/kg/day) significantly reduced the level of MDA in the liver of mice compared with CP-treated mice (*P*<0.001). The level of GSH decreased in the CP-treated animals compared with the control group (*P*<0.01). Pre-treatment of SA (5 mg/kg/day) with CP increased the GSH level compared with the CP alone group (*P*<0.05). 

***Histopathological findings***

Figure 4 illustrates changes in the histological architecture of the liver in all groups. The liver tissue structure (hepatocyte, Kupffer cells, and sinusoids) was normal in the control group (Figure 4A). SA at both doses of 5 and 10 mg/kg/day had no effect on the liver structure. The hepatocyte cords and the hepatic lobule were structurally normal and were similar to the untreated group (Figure 4B, C). The structure of the liver was destroyed in the CP-treated mice, and these pathologic changes such as degenerative changes in hepatocytes surrounding the central vein, inflammatory cell infiltration (thin black arrow), severe congestions, dilatation of sinusoids (thick black arrow), hemorrhage, cytoplasmic eosinophilic (white arrow), and vacuolation of hepatocytes were observed (Figure 4D). Administration of SA at both doses for 7 days in mice that received CP attenuated the structural destruction of the liver compared with the CP alone group (Figure 4E, F). However, a low dose of SA (5 mg/kg) was more effective and improved the liver damage better compared with the higher dose. 

Figure 4 shows the liver injury scoring system in all groups. CP increased the liver damage score (2.17±0.4). Administration of SA at both doses to CP-treated mice reduced liver injury scores (1.38±0.52 and 1.57±0.53, respectively) compared with the CP alone group (2.17±0.41). This reduction was significant for the 5 mg/kg dose (*P*<0.05).

Giant. 4 shows the liver injury scoring system in all groups. CP increased the liver damage score (2.17±0.4). Administration of SA at both doses to CP-treated mice reduced liver injury scores (1.38±0.52 and 1.57±0.53, respectively) compared with the CP alone group (2.17±0.41). This reduction was significant for the 5 mg/kg dose (*P*<0.05).

***Immunohistochemistry findings***

In the photomicrograph of the liver tissue in the immunohistochemical evaluation (Figures 5A and 6A), cells with increased immunoreactivity of apoptosis and inflammation were marked with brown color. Cells with positive immunoreactivity of caspase 3 and NF-κB were mostly found in liver cells adjacent to the central vein. Administration of synaptic acid at both doses was able to reduce the positive cells in mice receiving CP (Figures 5B and C, 6B and C).

As shown in Figure 7, when evaluated quantitatively, the expression levels of caspase 3 (Figure 7A) (*P*<0.0001) and NF-κB (Figure 7B) (*P*<0.0001) were increased in CP-treated mice compared with the untreated group. SA treatment at both doses significantly reduced caspase-3 and NF-κB immunoreactivity compared with mice that received CP alone (*P*<0.05).

**Table 1:** Levels of liver enzymes, aspartate aminotransferase (AST), alanine aminotransferase (ALT), and alkaline phosphatase (ALP)

Liver Enzymes	Control	SA5	SA10	CP	SA5+CP	SA10+CP
AST **(U/L)**	53±6.21	47±5.2	59±9.5	136.4±11.9^ aaaa^	104.8±13.7^ bbb^	122.6±12.3
ALT **(U/L)**	43.7±8	44.5±11.7	51.2±7.3	74±8.6^ aaaa^	56.7±8^ b^	61±7.6
ALP **(U/L)**	112.7±11.2	115.5±16.2	265.5±21.5	265.5±21.5^ aaaa^	200.7±14.5^ bbbb^	232.5±13.7^ bb^

All numbers are presented as mean ± SD. a, significant vs control; b, significant vs CP. cyclophosphamide (CP); Sinapic acid (SA)

**Figure 3:**
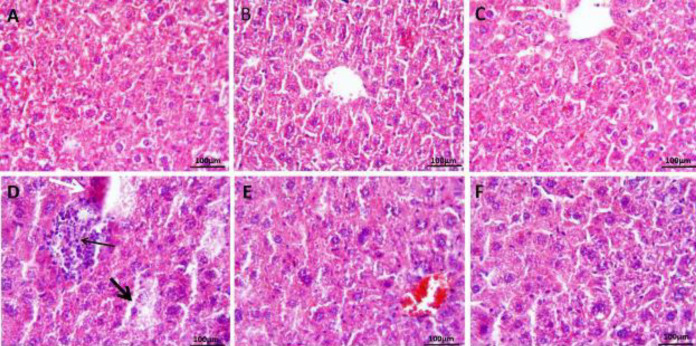
Micrographs from the histological structure of the liver. (A) Control, (B) SA (5 mg/kg), (C) SA with 10 mg/kg, have shown normal appearance in liver cells, (D) CP group has shown inflammatory cell infiltration (thin black arrow), severe congestions, dilatation of sinusoids (thick black arrow), hemorrhage, and cytoplasmic eosinophilic (white arrow), (E) SA (5 mg/kg) + CP, and (F) SA (10 mg/kg) + CP have shown mild inflammation and cell infiltration. H&E staining; 20× magnification; Scale bar = 100 μm. Cyclophosphamide (CP); sinapic acid (SA)

**Figure 4:**
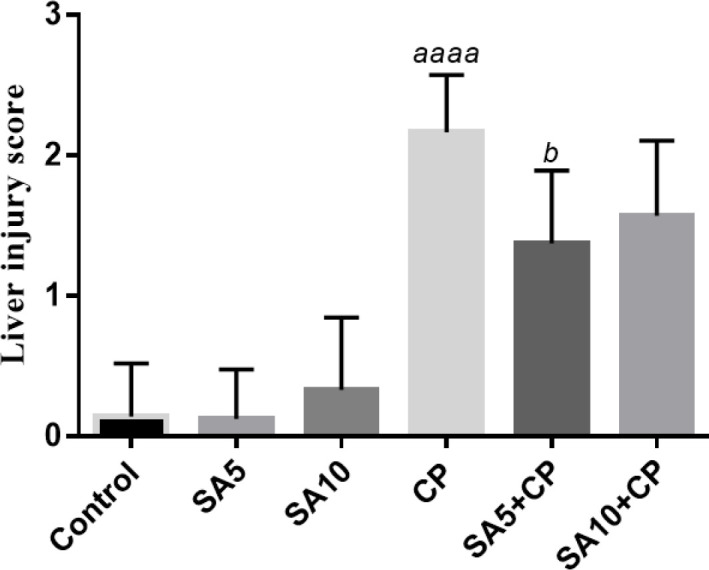
Semi-quantitative analysis in liver tissue. All charts are presented as mean ± SD. a, significant vs control; b, significant versus CP group. Cyclophosphamide (CP); sinapic acid (SA)

**Figure 5:**
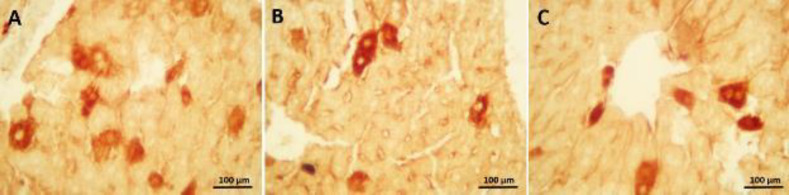
(a) Immunohistochemical test illustrated Caspase-3 immunoreaction in the CP group, which was considerable in the hepatocytes around the central vein. (b) SA (5 mg/kg) and (c) SA (10 mg/kg) treatment diminished Caspase-3 immunoreaction in CP-received mice. Cyclophosphamide (CP); sinapic acid (SA)

**Figure 6:**
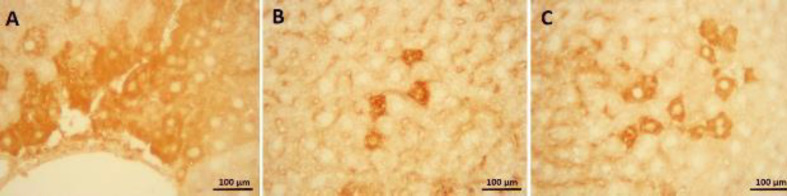
(a) Immunohistochemical test displayed NF-kB immunoreaction in the CP group, cells with brown color detectable around the central vein. (b) SA (5 mg/kg) and (c) SA (10 mg/kg) treatment alleviated NF-kB immunoreaction in CP-received mice. Cyclophosphamide (CP); Sinapic acid (SA)

**Figure 7:**
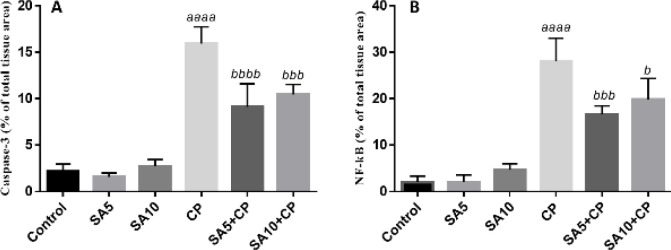
Liver damage scoring system for caspase-3 and NF-kB expression in the liver tissue. All charts are presented as mean ± SD. a, significant versus control and b, significant versus cyclophosphamide (CP); sinapic acid (SA)

## Discussion

CP is a chemotherapy agent that is widely used in cancer patients. Due to the side effects it has on most organs, its usefulness in the clinic is limited (18). Oxidative stress, inflammation, and apoptosis play significant roles in CP-induced hepatotoxicity (8). SA with anti-oxidant and anti-inflammatory potential could also have protective effects on CP-induced hepatotoxicity. In this study, the hepatoprotective effect of SA in CP-induced injury was evaluated using biomarkers of oxidative stress, and histological and immunoreactivity examinations. Changes in oxidative stress biomarkers demonstrated oxidative stress in CP-treated mice. Liver histological data were approved for elevated liver enzymes in the blood. SA reduced parameters of oxidative stress, apoptosis, and inflammation in CP-treated mice.

Many studies have demonstrated the effect of cyclophosphamide on biomarkers of oxidative stress as an intervening factor in the induction of liver damage (8, 19). The role of oxidative stress has been investigated by experiments such as MDA and GSH. In this study, MDA increased and GSH decreased in the liver of CP mice compared with the untreated group, indicating an increase in oxidative stress. An increase in MDA as a marker of lipid peroxidation and a decrease in GSH as an anti-oxidant marker have been reported in CP mice, leading to the induction of oxidative stress and subsequently hepatotoxicity (6, 20). The free radical scavenging activity of SA has been demonstrated by researchers (21). Administration of SA at both doses (5 and 10 mg/kg) significantly and dose-dependently attenuated oxidative stress. Ansari found that these results were consistent with our results (22). However, he used SA at 10 and 20 mg/kg, whereas in our pilot study, 20 mg/kg of SA induced liver tissue damage.

The liver is the main organ for the body’s metabolism in synthesis, catalysis, and evolution (23). Liver marker enzymes such as ALT, AST, and ALP increase in hepatotoxicity, necrosis, and inflammation of hepatocytes (24, 25). In this study, the elevation of liver enzymes confirmed liver tissue damage in mice treated with CP. The increase in ALT, AST, and ALP activities is likely due to the increased lipid peroxidation associated with CP which is followed by destruction of cell membrane integrity. Liver enzyme activities in mice receiving CP were reduced by SA treatment at both doses. Our results indicated that SA has a preventive role in CP-induced liver injury. 

In this study, the anti-inflammation property of SA was determined using an immunohistochemical assay by evaluating NF-κB in the liver. Data from this study showed increased NF-κB immunoreactivity in the CP-treated mice and SA-suppressed inflammation in CP-treated mice. Inflammation-related liver damage is mediated mostly by pro-inflammatory cytokines. Caglayan reported that TNF-α and IL-1β are increased in the body after cyclophosphamide administration. The expression of these cytokines is adjusted by NF-κB and has a role in the treatment of inflammatory disease (26). In oxidative stress-induced hepatotoxicity, NF-κB is activated in liver cells (hepatocytes and stellate cells), *et al.* and anti-oxidants are able to control these changes (27). Yun revealed that SA with NF-κB inactivation is able to inhibit inflammation (11). SA has also been found to attenuate liver injury through inhibition of NF-κB in tetrachloromethane-induced liver injury (14). Thus, SA treatment decreased NF-κB expression and inhibited inflammation. Our data are confirmed by this study, where SA with anti-oxidant activity inhibits inflammation (28).

The anti-apoptotic property of SA on liver tissues was specified by immunohistochemical assay in this study. Mice that received CP showed a significant increase in the apoptotic area in the liver. These areas were significantly reduced compared with SA-treated animals. SA suppressed caspase-3 immunoreactivity in the liver of CP-induced animals. In the same context, SA prevented gentamicin-induced apoptosis (29), cadmium-induced nephrotoxicity (30), and doxorubicin-induced cardiotoxicity (28) and protected human skin fibroblasts from UVB-induced skin photoaging (31). These studies were consistent with the results of our study showing SA reduced apoptosis by reducing oxidative stress. We assessed the role of SA in caspase-3 signaling. SA suppressing oxidative stress inactivates apoptosis marker expression (28). These data reveal that the anti-apoptotic activity of SA is responsible for the hepatoprotective property of SA in CP-induced hepatotoxicity in mice, a result that is reinforced by other investigations (32).

## Conclusion

We have shown that SA can be efficient in the recovery of liver injury induced by CP. SA repressed the immunoreactivity of caspase-3 and NF-κB by anti-oxidant activity. The data of this research acknowledged that SA could have a preventive effect on hepatotoxicity induced by CP.

## Authors’ Contributions

FTA and SJH participated in research design; SR and MM conducted experiments; FTA, MZ, and AK performed data analysis; FTA and SJH wrote or contributed to the writing of the manuscript. 

## Data Availability Statement

All data generated in this study are included in this manuscript.

## Conflicts of Interest

There are no conflicts of interest in this study and publication.
